# Descriptive Analysis of the Varroa Non-Reproduction Trait in Honey Bee Colonies and Association with Other Traits Related to Varroa Resistance

**DOI:** 10.3390/insects11080492

**Published:** 2020-08-01

**Authors:** Sonia E. Eynard, Christina Sann, Benjamin Basso, Anne-Laure Guirao, Yves Le Conte, Bertrand Servin, Lea Tison, Alain Vignal, Fanny Mondet

**Affiliations:** 1 GenPhySe, Université de Toulouse, INRAE, ENVT, 31320 Castanet-Tolosan, France; bertrand.servin@inrae.fr (B.S.); alain.vignal@inrae.fr (A.V.); 2 UMT PrADE, Protection des Abeilles dans l’environnement, 84914 Avignon, France; benjamin.basso@inrae.fr (B.B.); yves.le-conte@inrae.fr (Y.L.C.); lea.tison@inrae.fr (L.T.); fanny.mondet@inrae.fr (F.M.); 3 Labogena, 78350 Jouy-en-Josas, France; 4 ITSAP, 84914 Avignon, France; anne-laure.guirao@inrae.fr; 5 Abeilles et Environnement, INRAE Avignon, 84914 Avignon, France; 6 Santé et Agroécologie du Vignoble, INRAE Bordeaux, 33882 Villenave-d’Ornon, France

**Keywords:** *Apis mellifera*, *Varroa destructor*, mite non-reproduction (MNR), suppressed mite reproduction (SMR), varroa sensitive hygiene (VSH), hygienic behavior

## Abstract

**Simple Summary:**

Honey bees, *Apis mellifera*, are currently facing drastic colony losses, some of which lies partially on the infestation by a parasite, *varroa destructor*. To maintain healthy honey bee colonies breeding for resistance to this infestation seems promising. However, measuring resistance is a tedious task as it is a complex process. One can consider a honey bee colony resistant to varroa infestation when the parasite fails reproducing and thus its population does not grow in the colony, this is called mite non reproduction. In this study we dissected the performance of a scoring method for mite non reproduction and compared it to other known methods to measure resistance. Although the mite non reproduction method is preferred for field studies because of its relative simplicity it appears to lack correspondence with other methods and it should be interpreted cautiously because it is variable. Even though resistance to varroa infestation is desired for breeding decision it should be done carefully when the way to measure such resistance is using the mite non reproduction method.

**Abstract:**

In the current context of worldwide honey bee colony losses, among which the varroa mite plays a major role, the hope to improve honey bee health lies in part in the breeding of varroa resistant colonies. To do so, methods used to evaluate varroa resistance need better understanding. Repeatability and correlations between traits such as mite non-reproduction (MNR), varroa sensitive hygiene (VSH), and hygienic behavior are poorly known, due to practical limitations and to their underlying complexity. We investigate (i) the variability, (ii) the repeatability of the MNR score, and (iii) its correlation with other resistance traits. To reduce the inherent variability of MNR scores, we propose to apply an empirical Bayes correction. In the short-term (ten days), MNR had a modest repeatability of 0.4, whereas in the long-term (a month), it had a low repeatability of 0.2, similar to other resistance traits. Within our dataset, there was no correlation between MNR and VSH. Although MNR is amongst the most popular varroa resistance estimates in field studies, its underlying complex mechanism is not fully understood. Its lack of correlation with better described resistance traits and low repeatability suggest that MNR needs to be interpreted cautiously, especially when used for selection.

## 1. Introduction

Today, there is a common consensus that, while the origin of worldwide *Apis mellifera* colony losses is multifactorial, the parasite *Varroa destructor* contributes significantly to the weakening of honey bee populations [1,2]. The varroa mite is a honey bee parasite that affects bees by feeding on them while also transmitting potent viruses [3,4,5]. Currently, *A. mellifera* colonies, with only a few documented exceptions, are dependent on human intervention to survive mite infestations [1,6,7]. However, methods for controlling mite levels by means of acaricide treatments are losing effectiveness due to the ability of varroa to become resistant to certain molecules [8]. Furthermore, beekeepers struggle with the possibility that these chemicals can contaminate bee products such as honey or wax [9].

In the 1990s, *A. mellifera* colonies that do not require acaricide treatments to survive *V. destructor* infestation were discovered [10,11]. Since then, the existence of resistant *A. mellifera* populations has been confirmed in different regions of the world [12]. This has led to the hope, in the scientific community as well as among beekeepers, that an attractive sustainable long-term solution to counter the mite is the selection of honey bee populations that can naturally survive the parasite without the need for acaricide and regular human intervention [13]. In an *A. mellifera* colony, the varroa mite population size typically increases exponentially during the season due to the varroa foundresses being able to produce several fertile daughters during several reproductive cycles [14]. Contrary to the colonies that need treatment, surviving ones can support varroa mite infestation without a reduction in the longevity of the colony (tolerance) or can resist *V. destructor* by maintaining low varroa levels on their own (resistance) [15]. The selection and conservation of these colonies is, however, time consuming and a difficult task. To date, the success of breeding programs worldwide is low [16] and the commercial availability of resistant honey bee stock is rare. This is partly due to the difficulty of unambiguously identifying and understanding the resistant traits before being able to use them in dedicated selection programs.

One of the important traits identified was termed suppressed mite reproduction (SMR) [17]. It was first observed as a reduced reproductive output of foundresses in, for example, Africanized honey bees [18,19]. This trait depends on multiple mite or bee related factors. Firstly, the mites might already have a reduced fecundity when entering the brood cell [20,21]. Secondly, the brood itself can have an influence on the reproductive success of the varroa mite. Milani et al. [22] could show that molecules found in the brood cells can reduce the number of offspring produced by a varroa foundress. Lastly, the adult bees themselves can also reduce the reproductive success of the varroa mite by a behavior termed varroa sensitive hygiene (VSH) as well as by a recapping of infested cells [23]. The terminology SMR implies an active contribution of an external agent to the reproduction failure of varroa, even though such failure can be intrinsic to the varroa, as described above. This is why the new terminology, mite non-reproduction (MNR), has been recently proposed [24] and will be used thereafter in this study. Bees that express VSH can detect and remove a varroa-infested brood before the foundress can produce fertile daughter mites [25,26,27,28]. Recapping, a less costly option for the bees, is expressed by the uncapping of the infested brood by the nurse bees, followed by a subsequent recapping of the cell without harming the developing pupae [29,30,31]. So far, the outcome of selecting MNR colonies ranges from successfully identifying and selecting MNR colonies [12,32,33,34] to seeing no effect on the survival of honey bee colonies when looking at their MNR trait [35,36,37,38]. While it is possible to pin this on differences in the survival mechanisms used by distinct honey bee populations [12], it is also possible that this is at least partially due to methodological biases. Although there have been recommendations concerning how to perform the MNR measurement, they have changed over time [17,39], and different authors seem to use different research protocols, which makes it difficult to correctly compare their findings internationally.

We believe that the MNR measurement is indispensable to the continuation of comprehensive research on bee resistance to the varroa mite in the future as well as contributing to a successful and pertinent selection of resistant honey bees. However, we are in urgent need of a standard protocol to be used worldwide. Therefore, we aim in this study to validate an MNR protocol and point out the constraints and opportunities of this method to encourage its use in the future.

## 2. Materials and Methods

This study investigates (i) the variability of the MNR score (formerly SMR) obtained using the reference protocol, (ii) the repeatability of the MNR trait in comparison with other resistance traits, and (iii) the correlation between MNR and other resistance traits. For this purpose, a total of 275 honey bee colonies was used. All colonies were located in the facilities of Institut Technique et Scientifique de l’Apiculture et de la Pollinisation (ITSAP) and Institut National de Recherche pour l’Agriculture, l’alimentation et l’Environnement (INRAE) (Avignon, France); they were closely monitored for colony dynamics, varroa infestation, and varroa resistance behaviors from 2016 to 2019 (details in Table S1). All colonies were managed according to local beekeeping practices, including a yearly transhumance to the lavender fields between July and August. In order to infer the impact of varroa infestation and to be able to measure correlated varroa resistance-related traits, the colonies were left untreated for varroatosis during the course of the experiment or until their natural death. The colonies, originating from diverse sources, were not selected during the experiment and thus only experience natural selection. However, colonies were chosen to allow for varroa resistance inference, which means that some colonies entered the experiment because they had shown resistance or were expected to be resistant. The colonies’ genetic backgrounds were not controlled.

### 2.1. MNR Measurements

In this study, mite non-reproduction was determined by the absence of any viable daughter mites by the time the adult bee left the cell. MNR was measured following the COLOSS protocol for SMR scores [39]. One of the brood frames harboring enough cells containing brood with worker pupae (purple eyes stage or older, days 7 to 12) was taken from each colony, and broods infested by a single varroa foundress were dissected. The reproductive status, reproducing (R) or non-reproducing (NR), of the varroa foundress within each single-infested cell was inferred: mites that produced at least one viable daughter were considered reproducing (R) mites, while the others were considered non-reproducing (NR) mites (i.e., they could not produce any viable daughters by the time the adult bee left the cell). Three different NR cases can be identified: (i) foundresses that had not reproduced at all, (ii) foundresses that had produced only female offspring, and (iii) foundresses that had started to produce offspring too late for their daughter to reach maturity before the bee emerged from its cell. If a male was present, the reproductive status was inferred by observing the timing of the varroa reproductive cycle, when the brood was in the early stage (days 7 to 9), whereas when the pupae were in the later stage (after 9 days), the reproductive status could be inferred by looking for mature varroa offspring (presence of shedding in the brood cell). This measurement is ideally performed after the end of the honey production period, when varroa infestation is expected to peak. MNR was measured, at least once during the beekeeping season, for 231 colonies.

In order to evaluate the repeatability of the MNR assay, repeated measures of MNR were performed with two different time intervals: in the first set of measures (short-term repeatability), MNR was tested every 10 days for 30 days, thus three times at the end of the beekeeping season for 31 colonies; in the second set of measures (long-term repeatability), MNR was measured between one and five times across the beekeeping season, for 55 colonies.

### 2.2. Variance of MNR Measurements

The MNR measurement protocol can lead to a theoretical error of up to 20% in MNR scores [3]. To validate this theory and estimate the variance of MNR score on field data, we measured MNR from 60 to 101 cells infested by a single varroa foundress for 39 colonies. First, the MNR score was estimated from the first 35 dissected cells, as done usually over the normal course of the experiment (“MNR_first”). Second, a total MNR score was estimated based on all the dissected cells (“MNR_tot”). Finally, for all the colonies, 10, 35, and 50 cells were resampled randomly 100 times from the dissected cells to estimate MNR scores; 32 of these colonies had enough dissected cells to allow for a random resampling of 80 cells to estimate MNR scores.

### 2.3. Other Measures of Varroa Resistance and Colony Dynamics

#### 2.3.1. Hygienic behavior

Hygienic behavior was tested using the pin-test protocol [40,41]. This test consists in piercing 50 brood cells with a pin and counting the number of such pinned cells that are cleaned or under ongoing cleaning after six hours. The hygienic test (HYG) was conducted two to five times during the beekeeping season, on 139 colonies.

#### 2.3.2. Varroa Sensitive Hygiene

Varroa sensitive hygiene (VSH) was measured at the end of the beekeeping season by artificial infestation [40] in 26 colonies. For each colony tested, 30 freshly capped brood cells randomly positioned on a frame were artificially infested by one varroa foundress. To do so, each cell was carefully uncapped with a scalpel, a vigorous varroa mite (removed by from adult bees of donor colony using the sugar shake method) was placed inside the cell, which was then recapped and the frame returned to the tested colony for seven days. VSH was calculated as the proportion of artificially infested cells that are uncapped and cleaned (emptied) on the 8th day after artificial infestation.

### 2.4. Colony and Mite Monitoring

Colony dynamics were monitored using the ColEval method [42]. The number of bees, the number of open and capped brood cells, and the quantity of honey and pollen were estimated on average once a month across the whole beekeeping season (from March to October).

Varroa mite infestation and dynamic were measured in two different ways. First of all, varroa infestation in the brood was measured simultaneously to MNR scoring, as this measure produces an estimation of brood infestation. Secondly, phoretic varroa load was measured using the detergent method [40]; the number of varroa mites on adult bees was estimated and expressed as the number of mites per 100 bees. Phoretic varroa load was measured multiple times during the beekeeping season (March to October), on the day that ColEval was performed.

### 2.5. Statistical Analysis

All statistical analyses and visualizations were executed using R 3.6.2 [43].

#### 2.5.1. MNR Variance

MNR score minimum, mean, and maximum were estimated for the resampling of 10, 35, 50, and 80 cells infested by a single varroa foundress as well as for the first MNR score (corresponding to the first 35 cells dissected) and the total MNR score. Pooled standard deviation, for samples of identical sizes, for the resampled colonies was estimated as follows:

(1) $$\bar{s}=\sqrt{\frac{s_{1}^{2}+s_{2}^{2}+\ldots +s_{k}^{2}}{k}}$$

where *s_i_* is the sample standard deviation over *k* colonies.

Finally, the coefficient of variation (CV), for re-sampled colonies, was estimated as follows:

(2) $$CV_{k}=\frac{SD_{k}}{Mean_{k}}$$

The coefficient of variation (CV) is commonly used to assess the precision of an estimate as it is a ratio of deviation to the mean. This estimate was pooled across the *k* colonies, as for the standard deviation in Equation (1). Exponential regressions were fitted on the average CV for the MNR score. CVs were predicted, using the package *car* [44], for a number of dissected cells infested by a single varroa foundress, increasing from one to 150 in order to infer the minimum necessary number of cells infested by a single varroa foundress dissected to obtain CV of 10%, 5%, and 1%.

#### 2.5.2. MNR Score Correction

Bias in MNR score is highly dependent on the number of cells infested by a single varroa foundress that one can find on the selected brood frame. The COLOSS protocol recommends the dissection of a minimum of 35 such cells. Leaning towards the application of this protocol, we managed to score MNR on 35 cells or more (up to 49 cells) in 81% of the tested colonies. However, due to low infestation levels and/or low amounts of capped brood in some colonies, the MNR score was measured on between 34 and as little as one cell infested by a single varroa foundress for 19% of the frames (*n* = 82). To avoid discarding such data points, we applied an empirical Bayes correction, equation (3), as proposed by Mondet et al. 2020 [3].

(3) $$\hat{MNR}=\frac{nr}{c}\sim \;Beta\left(\alpha ,\beta \right)$$

with *c* as the number of dissected cells infested by a single varroa foundress, *nr* as the number of non-reproductive mites, and $\frac{nr}{c}$ being an estimator of MNR. A beta distribution was fitted to available observations using the package *MASS* [45]. Parameters alpha and beta were estimated by the Limited memory Broyden-Fletcher-Goldfarb-Shanno method (L-BFGS-B) method based on MNR scores available, ranging from, but not including, 0 and 1 (416 observations on 229 colonies). Observed values were corrected as follows:

(4) $$EB\_MNR=\frac{\alpha +nr}{\alpha +\beta +c}$$

Consequently, we estimated the Spearman rank correlation between raw MNR scores and empirical Bayes MNR scores, EB_MNR. Such a correction was applied throughout the rest of the analysis.

#### 2.5.3. Repeatability of Mite Resistance at Different Scales

Repeatability, considered as an estimation of the likelihood of obtaining multiple times the same result upon multiple evaluations of EB_MNR in a given colony, was estimated for short-term (multiple EB_MNR scores within a month) and long-term (multiple EB_MNR scores within a year) EB_MNR scores, as well as for hygienic behavior (HYG), as the following:

(5) $$R=\frac{V_{g}}{V_{p}}=\frac{V_{g}}{V_{g}+V_{e}}$$

where *V_g_* is the genetic variance, *V_e_* is the environmental variance, and *V_P_* is the phenotypic variance. In our case, *V_g_* was the colony variance and *V_e_* the residual variance estimated from a linear mixed-effect model.

#### 2.5.4. Correlations between EB_MNR and Other Resistance Traits

The cleaning of infested brood cells (VSH) was tested with an exact binomial test, with *p*-values adjusted for multiple testing by the Bonferroni correction. The correlation between VSH and EB_MNR was estimated using a Spearman rank correlation.

#### 2.5.5. Effects on Resistance Traits

The effects of variables linked to scoring period (year, month), location (apiary, county), beekeeper groups and queen origin, experimentation (observer), colony dynamics, and varroa infestation were tested for EB_MNR and HYG. For EB_MNR, nine qualitative and nine quantitative variables were tested using 446 observations on 231 colonies, and for HYG, five quantitative and seven qualitative variables were tested using 375 observations on 175 colonies. When testing EB_MNR and HYG, in order to obtain a complete dataset, missing data for the qualitative variables were set as “unknown” and missing data for the quantitative variables were imputed using the Factor Analysis of Mixed Data (FAMD) imputation function (with two principal components) from the *missMDA* package [46]. Quantitative variables were set as fixed effects while qualitative variables (scoring period, location, beekeepers and queen information, and observer) were set as random to control for structure in the population in the model. Fitting qualitative variables as random effects also allows for comparison between group effects within these qualitative variables in cases in which a large number of groups are represented each by a small number of data points, these groups not representing the full grouping possibilities in the population.

We performed a backward model reduction on the two datasets (function step from *lmerTest* package in R [47]), as suggested by Zuur et al. [48], by first selecting random effects, for EB_SMR and HYG, from a full model and then selecting fixed effects. Such model selection is based on the Akaike information criterion (AIC). The effects of the selected variables were estimated by a mixed-effect model analysis. The significance of the fixed effects was inferred using *p*-values from the model and the significance of the random effects was inferred using confidence intervals, computed with the confint function (*lme4* package in R [49]).

## 3. Results

### 3.1. Variance of the MNR Score

Variance of the MNR score (formerly SMR) was estimated by 100 resampling events, for 10, 35, 50 (*n* = 39), and 80 cells (*n* = 32) infested by a single varroa foundress. The MNR score based on the first 35 cells, equivalent to the scoring in the field throughout the experiment, ranged from 0.17 to 0.69, with a mean of 0.39 and a standard deviation of 0.14.

**Figure 1:**
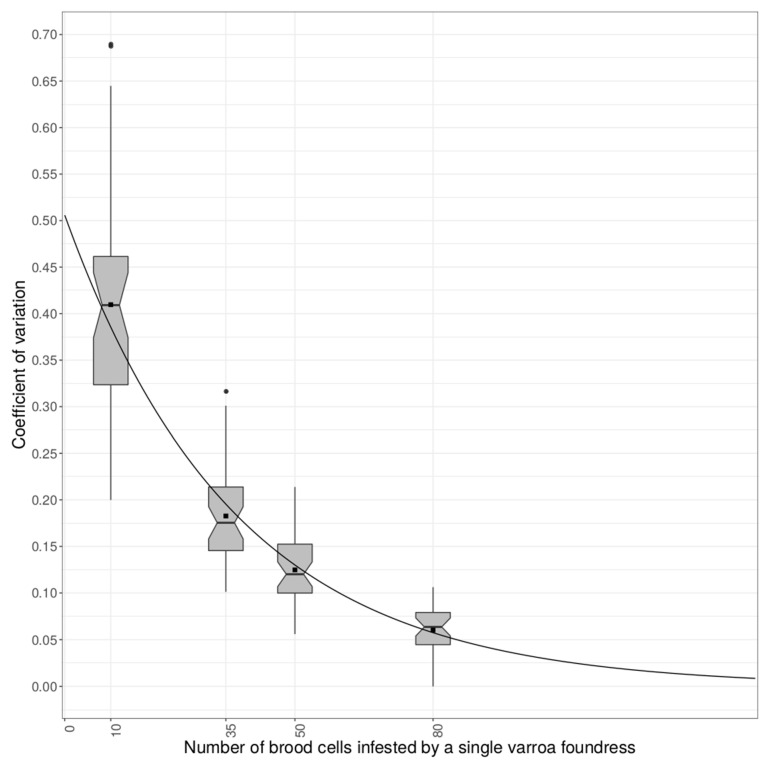
Boxplot of the coefficients of variation for the resampling events with 10, 35, 50, and 80 cells infested by a single varroa foundress. Each boxplot represents the range (black vertical line being the 95% interval range of the data) and the first and third quartiles (edge of the box); the horizontal line is the median value and the black square is the mean coefficient of variation (CV) value. Dots outside the boxplot are outlier values. The black line represents the exponential regression curve fitted to the data and allows the prediction of the minimum number of cells infested by a single varroa foundress necessary to reach 10% (60 cells), 5% (85 cells), and 1% (145 cells) variation for MNR.

The CV across colonies for the 100 resampling events was 42% when the MNR score was measured on 10 cells, 18% when measured on 35 cells, 12.8% when measured on 50 cells, and 6.0% when measured on 80 cells (Table 1 and Figure 1).

It is possible to predict the theoretical number of cells to be dissected to reach a specific variation by fitting an exponential regression to the average CV. If the aim is to reach a maximum of 10% variation—meaning that if the same colony is resampled multiple times, raw MNR scores will only vary by 10%—we inferred that at least 60 cells infested by a single varroa foundress were necessary to be dissected. To reach 5% and 1% of variation, the dissection of at least 85 and 145 cells infested by a single varroa foundress was, respectively, necessary (Figure 1).

### 3.2. MNR Score Correction

**Figure 2:**
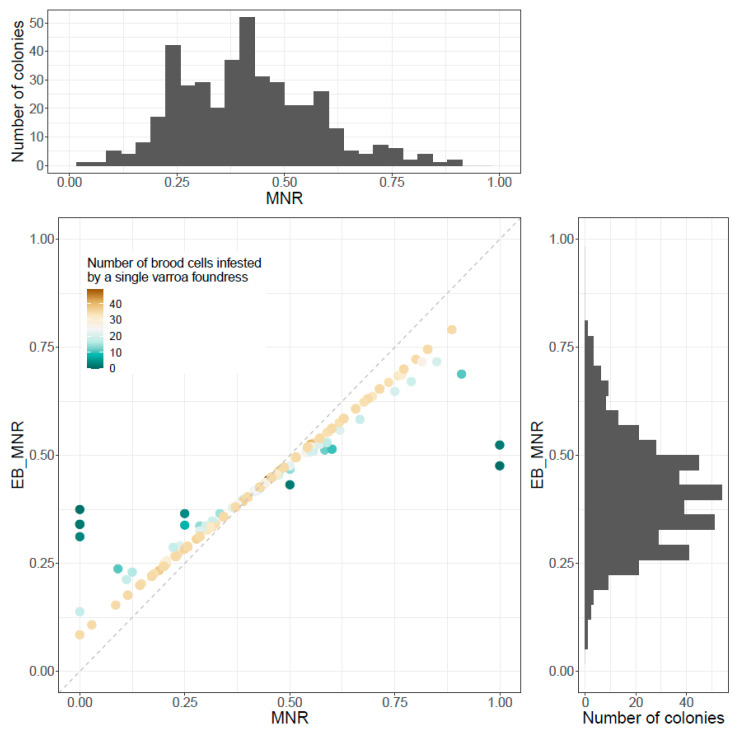
Scatterplot of the raw MNR score and the empirical Bayes EB_MNR score. Data points are colored according to the number of cells infested by a single varroa foundress dissected, dark green being close to zero and brown being up to more than 40. The histogram at the top represents the distribution, in number of colonies, of raw MNR score, and the histogram on the right, the distribution of EB_MNR score.

In order to avoid bias in MNR score due to a variable number of cells infested by a single varroa foundress dissected per brood frame, especially if estimates were based on a small number of dissected cells, an empirical Bayes correction was applied. Overall, raw MNR scores ranged from 0 to 1 when empirical Bayes MNR scores (EB_MNR) ranged from 0.08 to 0.79. Both raw MNR and EB_MNR have a mean of 0.41, which is expected when applying the empirical Bayes correction as it is equivalent to shrinkage of the data, therefore reducing the dispersion of the data but not affecting its mean. As expected when performing such corrections, the Spearman rank correlation between EB_MNR and raw MNR score was 0.99 (*p*-value < 10^−16^) (Figure 2). Thereafter, we used EB_MNR scores.

### 3.3. Repeatability of Mite Resistance Traits

Repeatability is a measure of the likelihood of obtaining multiple times the same result. The higher the repeatability, the more likely we are to obtain multiple times the same value—in our case, the same EB_MNR score.

#### 3.3.1. EB_MNR Repeatability

**Figure 3:**
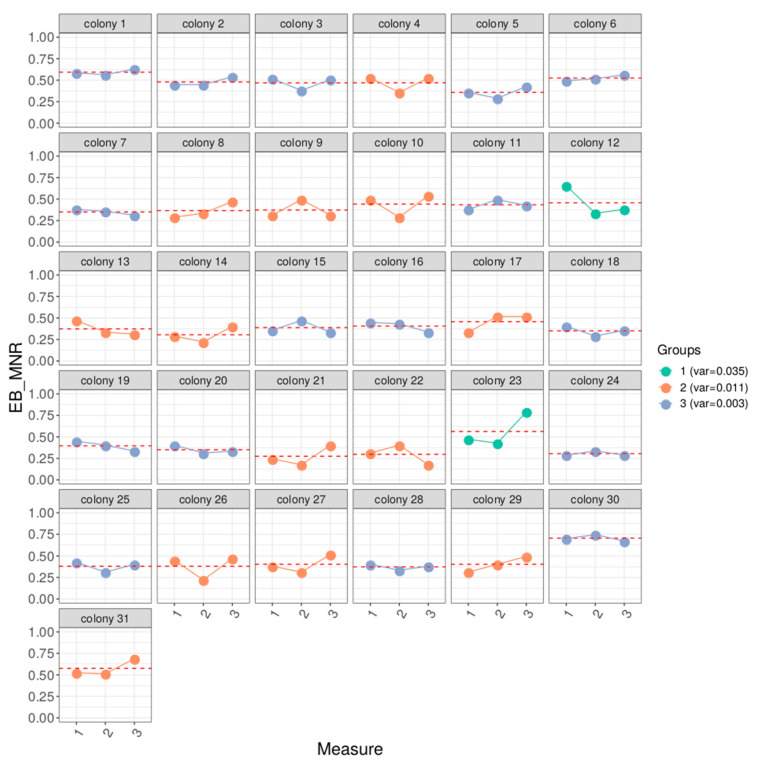
Short-term repeatability of MNR trait. EB_MNR score measured three times, once every 10 days, for each of the 31 colonies. The red dotted line represents the colony EB_MNR averaged across the three measures. The color indicates classification into one of the three variance groups: in green, the high variance group (two colonies), in orange, the medium variance group (13 colonies), and in blue, the low variance group (16 colonies).

EB_MNR short-term repeatability, as estimated by three measures once every 10 days, was 0.43 (standard error = 0.11, for 93 EB_MNR scores on 31 colonies). Intra-colony variance could be clustered, using k-means, in three groups having average variances of EB_MNR score of 0.034 (“high variability”, two colonies), 0.011 (“average variability”, 13 colonies), 0.003 (“low variability”, 16 colonies) (Figure 3). Long-term EB_MNR repeatability, over multiple measures within a year, was 0.17 (standard error = 0.09, for 148 EB_MNR scores on 55 colonies).

#### 3.3.2. Hygienic Behavior Repeatability

Hygienic behavior ranged between 0 and 1, with a mean of 0.74. Hygienic behavior measured multiple times within a year had a repeatability of 0.21 (standard error = 0.07, 339 measures of hygienic behavior on 139 colonies).

### 3.4. Correlations between Mite Resistance Traits

**Figure 4:**
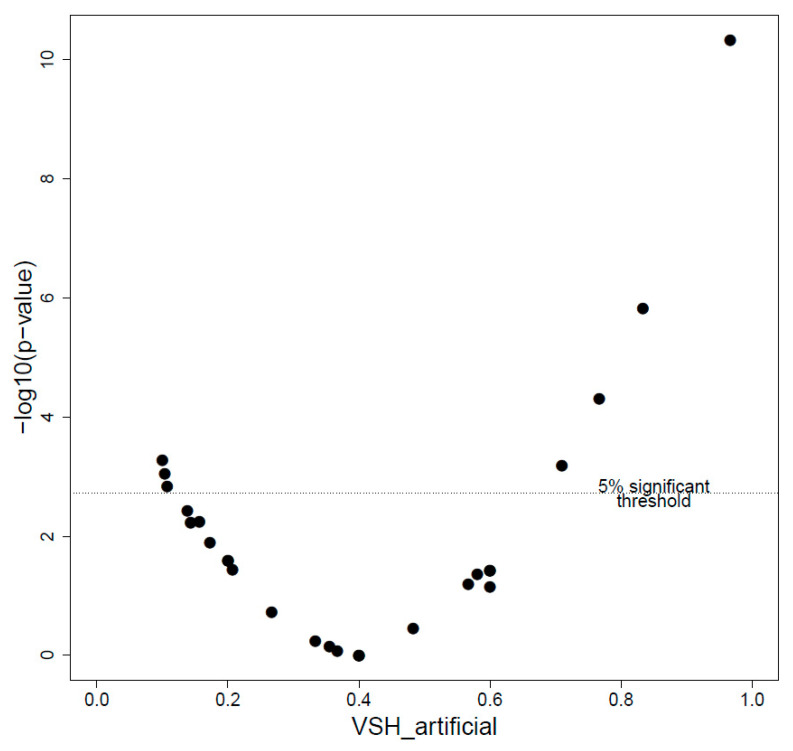
Scatterplot of the −log10 (*p*-values) for varroa sensitive hygiene (VSH). The dotted line represents the 5% significance threshold, in −log10, after Bonferroni correction for multiple tests. Points above the threshold line are colonies with significantly lower (to the left) or higher (to the right) cleaning rates than expected under the null hypothesis.

VSH, measured by artificial infestation on 26 colonies, ranged between 0.10 to 0.97 cleaning rate, with a mean of 0.40 and a median 0.36. Three colonies had a significantly low VSH, meaning that less brood cells were cleaned than expected under the null hypothesis of 0.40 cleaning (the mean VSH score of the tested colonies), whereas four colonies had a significantly higher VSH, meaning that more brood cells were cleaned than expected under the null hypothesis (Figure 4).

Finally, we estimated the Spearman rank correlation between EB_MNR (a unique measure per colony) and VSH, as both measures were made for the colonies of interest on the same date, while on the same apiary and after the exact same treatment throughout the experiment (transhumance at the same date, same beekeeping practices). Having the two measures scored in the same conditions enables direct comparisons free from external effects. There were no correlations between EB_MNR and VSH (25 colonies, Spearman rank correlation = 0.22, *p*-value = 0.29) (Figure S1).

### 3.5. Effects on Resistance Traits

#### 3.5.1. EB_MNR

Variables retained as random effects in the best model were scoring period, breeder’s group, and the observer, while variables retained as fixed effects were the amount of honey in the hive and the recapping behavior of the colony. Overall EB_MNR was significantly influenced by the random effects of scoring period and breeder’s group, with the breeder’s group INRAE, having “survivor” colonies kept for at least three years [50], presenting the highest effect and showing the highest EB_MNR values. The trait EB_MNR was also significantly influenced by both fixed effects—amount of honey in the hive (negative effect) and recapping behavior of the colony (positive effect) (Table 2) — meaning that little honey and high recapping behavior were associated with high EB_MNR scores.

#### 3.5.2. Hygienic Behavior

Variables retained as random effects in the best model were queen’s genetic origin and the testing apiary, while the only variable retained as a fixed effect was the phoretic varroa infestation level, being the only one linked to varroa infestation level in the colony available for HYG measure. Overall, HYG was significantly influenced by the testing apiary and the varroa infestation. The fixed effect linked to varroa infestation was negative (Table 3), meaning the higher the varroa infestation, the lower the HYG score.

## 4. Discussion

In this study, we aimed at describing the MNR, more pertinent than the previously known SMR, as the current protocols lead to a large variation in MNR scores. This variation can be lessened by increasing the number of dissected cells or, if this does not lie in the scope of the study, by applying an empirical Bayes correction. MNR short-term repeatability was larger than long-term repeatability, the latter being similar to the repeatability of hygienic behavior. Finally, no correlation between MNR and VSH could be observed in our dataset. This in-depth analysis of mite non-reproduction (MNR) highlights several points that need to be considered when using this trait for experiments and breeding efforts.

The current guidelines proposed by COLOSS [39] for MNR measurement advises on a minimum of 35 cells infested by a single varroa foundress to be dissected, for which mite reproduction should be analyzed per colony [39]. When using such guidelines, we reported around an 18% variation for raw MNR. This suggests that estimates relying on 35 cells or less can potentially be unreliable. For instance, when the goal of an experiment is to sample for colonies with an MNR of at least 60%, while relying on a 35-cell estimate, only colonies with an MNR of at least 71% are guaranteed to fit the criteria. Low precision observed for the MNR score supports theoretical findings from Mondet et al. [3], where it was shown that 35 cells is the minimum target to obtain relatively reliable MNR scores. This needs to be considered especially for scientific studies in which case rankings have to be performed with extreme care. If the interest lies in exclusively examining high values, as can be done for breeding efforts, sampling 35 or less cells may be sufficient to select colonies, as shown in [20]. In fact, care should be taken regarding the threshold used for selection and it should be tightened when a smaller number of cells are dissected to estimate MNR.

Questions arose especially for colonies with a small number of cells infested by a single varroa foundress. These colonies have the potential to be particularly interesting, as the low varroa load could point to a resistance to infestation. Two approaches can be used to deal with such data: one is to set a cut-off for the number of cells infested by a single varroa foundress necessary to validate the subsequent MNR score and the other is to apply an empirical Bayes correction to the raw MNR scores, leading to shrinkage in MNR score distribution. The first strategy is likely to be preferred by professional beekeepers due to its simplicity, whereas the second might be preferred for research purposes as it avoids the application of arbitrary thresholds. However, both lead to the rejection of the colonies with extreme MNR scores, one by removal and the second by fading due to the shrinkage. This means that potentially resistant colonies are not being taken into account because they cannot be properly evaluated due to a low varroa load. One solution could be to artificially control the hive varroa infestation. However, this could cause bias in the MNR scoring procedure. To date, there has been no strategy developed to counter this bias; we recommend future studies to focus on estimating sampling bias by dissecting an extensive number of cells, ideally full frames without applying cut-off or by carefully combining MNR trait with other traits of the colony. However, the development of such an index relies on a thorough understanding of the different traits to combine and their interactions.

The results presented here also highlight the fact that short-term repeatability (ten days’ time intervals) of MNR was modest, whereas long-term repeatability (within years) measurements were relatively low, although comparable to those of hygienic behavior [51,52], a main trait used as a selection criterion in several breeding programs. Measuring hygienic behavior has successfully led to the selection of hygienic honey bees [53], meaning that, nevertheless, there is potential for MNR to be used for selection if its heritability is high enough, which still has to be confirmed.

The low repeatability may be partially explained by the variance of estimates described above (i.e., values potentially may be up to 18% too high or too low when using 35 cells), meaning that the resistance score of a colony could be much more congruent than what we see in our dataset. A shortcoming of our study is the lack of consideration of environmental variables. However, it is known that environmental variables such as temperature and humidity can potentially affect resistant traits [54,55], with temperature being negatively and humidity being positively correlated to the infestation growth rate of the mite [56]. Resource availability is also known to influence hygienic behavior [57,58], as well as task repartition in the colony, which may also affect the MNR trait. For instance, during a period with strong nectar flow, as experienced by our colonies in July, there may be trade-offs between brood care and foraging and the expression of the VSH trait. Evaluation at the end of August can thus be influenced by the low proportion of brood in the colony versus stored honey (Tison et al., personal communication). Furthermore, resistance traits can be biased by the horizontal transmission of varroa mites by the drifting of bees or the robbing of hives [59,60,61], especially if the amount of transferred mites differs between colonies of the same apiary [16]. Additionally, colony and mite dynamics are highly changing through time and may influence mite reproduction and therefore MNR results. In our study, we accounted for colony dynamics, colony management, and location of the hives, none of which significantly impacted MNR. Mite infestation in the brood cells and on adult bees did not affect the MNR trait either. This corresponds with the observations of different authors, stating that the link between MNR and mite infestation levels is not universal [3,35,62,63].

All of the above demonstrate that MNR is a complex mechanism combining multi-factorial effects, such as adult bee behavior, brood and mite physiology, and bee and mite genetics. There is an urgent need for further analysis to disentangle potential environmental effects on the MNR mechanism. We can also assert that experimental design is one of the major limitations to drawing solid conclusions. The lack of balance in the experimental design can lead to spurious effect estimation and interpretations. Controlling the experimental design is somehow difficult in the field but should be evaluated upstream at the research facilities. Lastly, even though we could observe significant differences between VSH across our colonies, contrary to what has been previously found by Harbo and Harris [25], no correlation between MNR and VSH (varroa sensitive hygiene) were observed in our dataset. However, it is known that varroa resistance mechanisms can differ between populations and that resistance traits are not always informative of resistance to varroa infestation in unselected populations [64]. It thus seems legitimate to genetically distinguish each population and investigate its unique resistance behavior [65]. Moreover, it has been shown that the selection of resistance traits is potentially challenging because of parasite adaptation. As observed earlier [66], it is possible that, even though some colonies harbor significantly higher VSH scores, selecting them might not lead to a perennial selection for many generations. We could expect the same to be valid for MNR, making the use of this resistance trait difficult in practice.

## 5. Conclusions

In conclusion, the MNR measurement remains one of the few measurements for varroa resistance in honey bee populations, which can be achieved in the field on a relatively large scale. Although time consuming and tedious to implement, it also gives a lot of different information which can help us to better understand the control mechanisms that bees use to counteract the varroa mite. However, the results here highlight the need for a precise protocol using enough single infested cells (>35), performed multiple times over a short period of time to provide solid estimates. The weak points should be taken into consideration when designing an experiment, and a combination of different measurements to correctly assess honey bee resistance like mite infestation levels (inside and outside the brood cells) and genetic analysis could be additionally taken into account when analyzing the varroa resistance of a colony. To date, no breeding programs aiming at obtaining resistant honey bees have produced commercially available colonies. We believe that using the MNR measurement with a new awareness of its weaknesses and strengths could be an important tool for successful future selection programs of resistant honey bees.

## Figures and Tables

**Table 1:** Summary table containing the minimum, mean, maximum, standard deviation, and coefficient of variation for the first (MNR_first) and total mite non reproduction (MNR) (MNR_tot). For the 10 (MNR_10), 35 (MNR_35), 50 (MNR_50), and 80 (MNR_80) resampling events, minimum, mean, maximum, as well as pooled standard deviation and pooled coefficients of variation with their 95% confidence interval, are reported.

	Minimum	Mean	Maximum	Standard Deviation	Coefficient of Variation
MNR_first (35 cells) (*n* = 39)	0.171	0.394	0.686	0.141	0.357
MNR_tot (*n* = 39)	0.165	0.373	0.680	0.123	0.331
MNR_10 (*n* = 39 × 100)	0.000	0.370	1.000	0.142 [0.138; 0.145]	0.420 [0.380; 0.460]
MNR_35 (*n* = 39 × 100)	0.000	0.374	0.886	0.062 [0.060; 0.064]	0.180 [0.164; 0.197]
MNR_50 (*n* = 39 × 100)	0.080	0.373	0.820	0.044 [0.041; 0.046]	0.128 [0.115; 0.141]
MNR_80 (*n* = 32 × 100)	0.125	0.356	0.725	0.020 [0.017; 0.022]	0.060 [0.051; 0.070]

**Table 2:** Summary table of the best model for EB_MNR. Standard deviations and 95% confidence intervals (to determine significance) are presented for the selected random effects. Coefficients, standard errors, degrees of freedom, and *p*-values are presented for the selected fixed effects.

**Random Effects**	**Standard Deviation**	**95% Confidence Interval**		
Colony	0.044	[0.024; 0.058]		
Breeders group	0.034	[0.004; 0.054]		
Scoring period	0.036	[0.014; 0.057]		
Observer	0.030	[0.000; 0.050]		
Residuals	0.090	[0.083; 0.099]		
**Fixed Effects**	**Coefficient**	**Standard Error**	**Degree of Freedom**	***p*-value**
Intercept	0.395	0.018	19.560	<10^−15^
Number recapped cells	0.015	0.005	420.554	0.007
In hive honey	−0.022	0.006	257.714	0.001

**Table 3:** Summary table of the best model for hygienic behavior (HYG). Standard deviations and 95% confidence intervals (to determine significance) are presented for the selected random effects. Coefficients, standard errors, degrees of freedom, and *p*-values are presented for the selected fixed effects.

**Random Effects**	**Standard Deviation**	**95% Confidence Interval**		
Colony	0.075	[0.000; 0.116]		
Queen’s genetic origin	0.069	[0.000; 0.107]		
Testing apiary	0.087	[0.032;0.139]		
Residuals	0.210	[0.190; 0.230]		
**Fixed Effects**	**Coefficient**	**Standard Error**	**Degree of Freedom**	***p*-value**
Intercept	0.731	0.034	8.0.38	<10^−8^
Phoretic varroa infestation	−0.042	0.012	305.427	5 × 10^−4^

## Supplementary Materials

The following are available online at https://www.mdpi.com/2075-4450/11/8/492/s1, Figure S1: Scatterplot of VSH for EB_MNR values for the 26 colonies used to estimate correlation between these traits. Table S1: Number of colonies in each of the different experiments and number of colonies additionally contributing to other experiments.

## References

[1] Boecking O., Genersch E. (2008). Varroosis—The Ongoing Crisis in Bee Keeping. J. Consum. Prot. Food Saf..

[2] Le Conte Y., Ellis M., Ritter W. (2010). Varroamites and honey bee health: Can varroa explain part of the colony losses?. Apidologie.

[3] Mondet F., Parejo M., Meixner M.D., Costa C., Kryger P., Andonov S., Servin B., Basso B., Bienkowska M., Bigio G. (2020). Evaluation of suppressed mite reproduction (SMR) reveals potential for varroa resistance in European honey bee (*Apis mellifera* L.). Insects.

[4] Wilfert L., Long G., Leggett H.C., Schmid-Hempel P., Butlin R.K., Martin S.J.M., Boots M. (2016). Deformed wing virus is a recent global epidemic in honeybees driven by Varroa mites. Science.

[5] Martin S.J., Kryger P. (2002). Reproduction of Varroa destructor in South African honey bees: Does cell space influence Varroa male survivorship?. Apidologie.

[6] Sammataro D., Gerson U., Needham G. (2000). Parasitic Mites of Honey Bees: Life History, Implications, and Impact. Annu. Rev. Èntomol..

[7] Dainat B., Evans J.D., Chen Y.P., Gauthier L., Neumann P. (2012). Predictive Markers of Honey Bee Colony Collapse. PLoS ONE.

[8] Genersch E., Von Der Ohe W., Kaatz H., Schroeder A., Otten C., Büchler R., Berg S., Ritter W., Mühlen W., Gisder S. (2010). The German bee monitoring project: A long term study to understand periodically high winter losses of honey bee colonies. Apidologie.

[9] Bajuk B.P., Babnik K., Snoj T., Milčinski L., Ocepek M.P., Škof M., Jenčič V., Filazi A., Štajnbaher D., Kobal S. (2017). Coumaphos residues in honey, bee brood, and beeswax after Varroa treatment. Apidologie.

[10] Le Conte Y., De Vaublanc G., Crauser D., Jeanne F., Rousselle J.-C., Bécard J.-M. (2007). Honey bee colonies that have survivedVarroa destructor. Apidologie.

[11] Mattila H.R., Seeley T.D. (2007). Genetic Diversity in Honey Bee Colonies Enhances Productivity and Fitness. Science.

[12] Locke B. (2015). Natural Varroa mite-surviving Apis mellifera honeybee populations. Apidologie.

[13] Neumann P., Blacquière T. (2016). The Darwin cure for apiculture? Natural selection and managed honeybee health. Evol. Appl..

[14] Calis J.N., Fries I., Ryrie S.C. (1999). Population modelling of Varroa jacobsoni Oud. Apidologie.

[15] Kurze C., Routtu J., Moritz R. (2016). Parasite resistance and tolerance in honeybees at the individual and social level. Zoology.

[16] Guichard M., Dietemann V., Neuditschko M., Dainat B. (2020). Three Decades of Selecting Honey Bees that Survive Infestations by the Parasitic Mite Varroa destructor: Outcomes, Limitations and Strategy. Preprint.

[17] Harbo J.R., Harris J.W. (2002). Suppressing Mite Reproduction: SMR an Update. Bee Cult..

[18] Mondragón L., Spivak M., Vandame R. (2005). A multifactorial study of the resistance of honeybees Apis mellifera to the mite Varroa destructor over one year in Mexico. Apidologie.

[19] Wendel H.P., Rosenkranz P. (1990). Invasionsgeschwindigkeit und Fertilität von Varroa-Weibchen in aufeinanderfolgenden Reproduktionszyklen. Apidologie.

[20] Harbo J.R., Harris J.W. (1999). Heritability in Honey Bees (Hymenoptera: Apidae) of Characteristics Associated with Resistance to Varroa jacobsoni(Mesostigmata: Varroidae). J. Econ. Èntomol..

[21] Fuchs S. (1994). Non-reproducing Varroa jacobsoni Oud. in honey bee worker cells?status of mites or effect of brood cells?. Exp. Appl. Acarol..

[22] Milani N., Della Vedova G., Nazzi F. (2004). (Z)-8-Heptadecene reduces the reproduction of Varroa destructor in brood cells. Apidologie.

[23] Oddie M., Dahle B., Neumann P. (2018). Reduced Postcapping Period in Honey Bees Surviving Varroa destructor by Means of Natural Selection. Insects.

[24] Mondet F., Beaurepaire A., McAfee A., Locke B., Alaux C., Blanchard S., Danka B., Le Conte Y., Fanny M., Alexis B. (2020). Honey bee survival mechanisms against the parasite Varroa destructor: A systematic review of phenotypic and genomic research efforts. Int. J. Parasitol..

[25] Harbo J.R., Harris J.W. (2005). Suppressed mite reproduction explained by the behaviour of adult bees. J. Apic. Res..

[26] Harris J.W., Danka R.G., Villa J.D. (2010). Honey Bees (Hymenoptera: Apidae) With the Trait of Varroa Sensitive Hygiene Remove Brood with All Reproductive Stages of Varroa Mites (Mesostigmata: Varroidae). Ann. Èntomol. Soc. Am..

[27] Kirrane M.J., De Guzman L.I., Whelan P.M., Frake A.M., Rinderer T.E. (2018). Evaluations of the Removal of Varroa destructor in Russian Honey Bee Colonies that Display Different Levels of Varroa Sensitive Hygienic Activities. J. Insect Behav..

[28] Kirrane M.J., De Guzman L.I., Holloway B., Frake A.M., Rinderer T.E., Whelan P.M. (2015). Phenotypic and Genetic Analyses of the Varroa Sensitive Hygienic Trait in Russian Honey Bee (Hymenoptera: Apidae) Colonies. PLoS ONE.

[29] Danka R.G., Harris J.W., Dodds G.E. (2015). Selection of VSH-derived “Pol-line” honey bees and evaluation of their Varroa-resistance characteristics. Apidologie.

[30] Oddie M., Büchler R., Dahle B., Kovačić M., Le Conte Y., Locke B., De Miranda J.R., Mondet F., Neumann P. (2018). Rapid parallel evolution overcomes global honey bee parasite. Sci. Rep..

[31] Martin S.J., Brettell L. (2019). Deformed Wing Virus in Honeybees and Other Insects. Annu. Rev. Virol..

[32] Harbo J.R., Harris J.W. (2001). Resistance to Varroa destructor (Mesostigmata: Varroidae) when mite-resistant queen honey bees (Hymenoptera: Apidae) were free-mated with unselected drones. J. Econ. Èntomol..

[33] Harbo J.R., Harris J.W. (2003). An Evaluation of Commercially Produced Queens That Have the SMR Trait. Am. Bee J..

[34] Buchegger M., Buechler R., Fuerst-Waltl B., Kovačić M., Willam A. (2018). Relationships between resistance characteristics of honey bees (Apis mellifera) against Varroa mites (Varroa destructor). J. Central Eur. Agric..

[35] Büchler R., Drescher W. (1990). Variance and Heritability of the Capped Developmental Stage in European Apis Mellifera L. and Its Correlation with Increased Varroa Jacobsoni Oud. Infestation. J. Apic. Res..

[36] Lodesani M., Crailsheim K., Moritz R. (2002). Effect of some characters on the population growth of mite Varroa jacobsoni in Apis mellifera L colonies and results of a bi-directional selection. J. Appl. Èntomol..

[37] Ibrahim A., Reuter G.S., Spivak M. (2007). Field trial of honey bee colonies bred for mechanisms of resistance againstVarroa destructor. Apidologie.

[38] Ibrahim A., Spivak M. (2005). The relationship between hygienic behavior and suppression of mite reproduction as honey bee (Apis mellifera) mechanisms of resistance toVarroa destructor. Apidologie.

[39] Büchler R., Costa C., Mondet F., Kezic N., Kovacic M. New Smr Protocol. https://www.beebreeding.net/index.php/2017/09/01/n.

[40] Dietemann V., Nazzi F., Martin S.J., Anderson D.L., Locke B., Delaplane K.S., Wauquiez Q., Tannahill C., Frey E., Ziegelmann B. (2013). Standard methods for varroa research. J. Apic. Res..

[41] Andere C., Palacio M., Rodriguez E., Dominguez M.T., Figini E., Bedascarrasbure E. (2000). Evaluation of honey bee defensive behavior in Argentina—A field method. Am. Bee J..

[42] Hernandez J., Maisonnasse A., Cousin M., Beri C., Le Quintrec C., Bouetard A., Castex D., Decante D., Servel E., Buchwalder G. (2020). ColEval: Honeybee COLony Structure EVALuation for Field Surveys. Insects.

[43] The R Project for Statistical Computing. http://www.r-project.org/.

[44] Fox J., Weisberg S. (2019). An R Companion to Applied Regression.

[45] Venables W.N., Ripley B.D. (2002). Modern Applied Statistics with S.

[46] Josse J., Husson F. (2016). missMDA: A Package for Handling Missing Values in Multivariate Data Analysis. J. Stat. Softw..

[47] Kuznetsova A., Brockhoff P.B., Christensen R.H.B. (2017). lmerTest Package: Tests in Linear Mixed Effects Models. J. Stat. Softw..

[48] Zuur A.F., Ieno E.N., Walker N., Saveliev A.A., Smith G.M. (2009). Mixed Effects Modelling for Nested Data.

[49] Bates D., Mächler M., Bolker B., Walker S. (2015). Fitting Linear Mixed-Effects Models Using lme. J. Stat. Softw..

[50] Le Conte Y., Mondet F. (2017). Natural Selection of Honeybees Against Varroa destructor. Beekeeping—From Science to Practice.

[51] Facchini E., Bijma P., Pagnacco G., Rizzi R., Brascamp E.W. (2019). Hygienic behaviour in honeybees: A comparison of two recording methods and estimation of genetic parameters. Apidologie.

[52] Maucourt S. Genetic Selection of the Honey Bee (Apis mellifera) in a Northern Climate. Proceedings of the 46th Apimondia International Apicultural Congress.

[53] Rinderer T.E., Harris J.W., Hunt G.J., De Guzman L.I. (2010). Breeding for resistance toVarroa destructorin North America. Apidologie.

[54] Kulincevic J.M., Rinderer T.E., Urošević D.J. (1988). Seasonality and Colony Variation of Reproducing and Non-Reproducing Varroa Jacobsoni Females in Western Honey Bee (Apis Mellifera) Worker Brood. Apidologie.

[55] Bienefeld K., Radtke J., Zautke F. (1995). Einfluss der Temperaturregulierung im Bienenvolk auf den Reproduktionserfolg von Varroa jacobsoni Oud. Apidologie.

[56] Harris J.W., Villa J.D., Danka R.G. (2004). Environmental effects on the growth of varroa mite populations. Bee Cult..

[57] Büchler R. (1994). Varroa tolerance in honey bees—Occurrence, characters and breeding. Bee World.

[58] Momot J.P., Rothenbuhler W.C. (1971). Behaviour Genetics of Nest Cleaning in Honeybees. VI. Interactions of Age and Genotype of Bees, and Nectar Flow. J. Apic. Res..

[59] Greatti M., Milani N., Nazzi F. (1992). Reinfestation of an acaricide-treated apiary byVarroa jacobsoni Oud. Exp. Appl. Acarol..

[60] Frey E., Rosenkranz P. (2014). Autumn invasion rates of Varroa destructor (Mesostigmata: Varroidae) into honey bee (Hymenoptera: Apidae) colonies and the resulting increase in mite populations. J. Econ. Èntomol..

[61] Peck D.T., Smith M.L., Seeley T.D. (2016). Varroa destructor Mites Can Nimbly Climb from Flowers onto Foraging Honey Bees. PLoS ONE.

[62] Weller S. (2008). Populationsdynamik der Parasitischen Bienenmilbe Varroa Destructor in Vorselektierten Bienenvölkern (A. mellifera L.) unter Besonderer Berücksichtigung der Reproduktion. Master’s Thesis.

[63] Ward K., Danka R., Ward R. (2008). Comparative performance of two mite-resistant stocks of honey bees (Hymenoptera: Apidae) in Alabama beekeeping operations. J. Econ. Entomol..

[64] Leclercq G., Pannebakker B., Gengler N., Nguyen B.K., Francis F. (2017). Drawbacks and benefits of hygienic behavior in honey bees (Apis mellifera L.): A review. J. Apic. Res..

[65] Wagoner K., Spivak M., Rueppell O. (2018). Brood Affects Hygienic Behavior in the Honey Bee (Hymenoptera: Apidae). J. Econ. Èntomol..

[66] Villa J.D., Danka R.G., Harris J.W. (2017). Repeatability of measurements of removal of mite-infested brood to assess Varroa Sensitive Hygiene. J. Apic. Res..

