# Supplementary material for: Descriptive Analysis of the Varroa Non-Reproduction Trait in Honey Bee Colonies and Association with Other Traits Related to Varroa Resistance
Source: Insects. 2020 Aug 1;11(8):492. doi: 10.3390/insects11080492 (PMC7469219; doi:10.3390/insects11080492)
Supplement: Supplementary file 1 [file insects-11-00492-s001.pdf]

Figure S1: Scatterplot of VSH for EB_MNR values for the 26 colonies used to estimate correlation between these traits. Red points are the colonies with (significantly) high VSH and the blue points with (significantly) low VSH values. The histograms represent the distribution, in number of colonies, of VSH and EB_MNR.

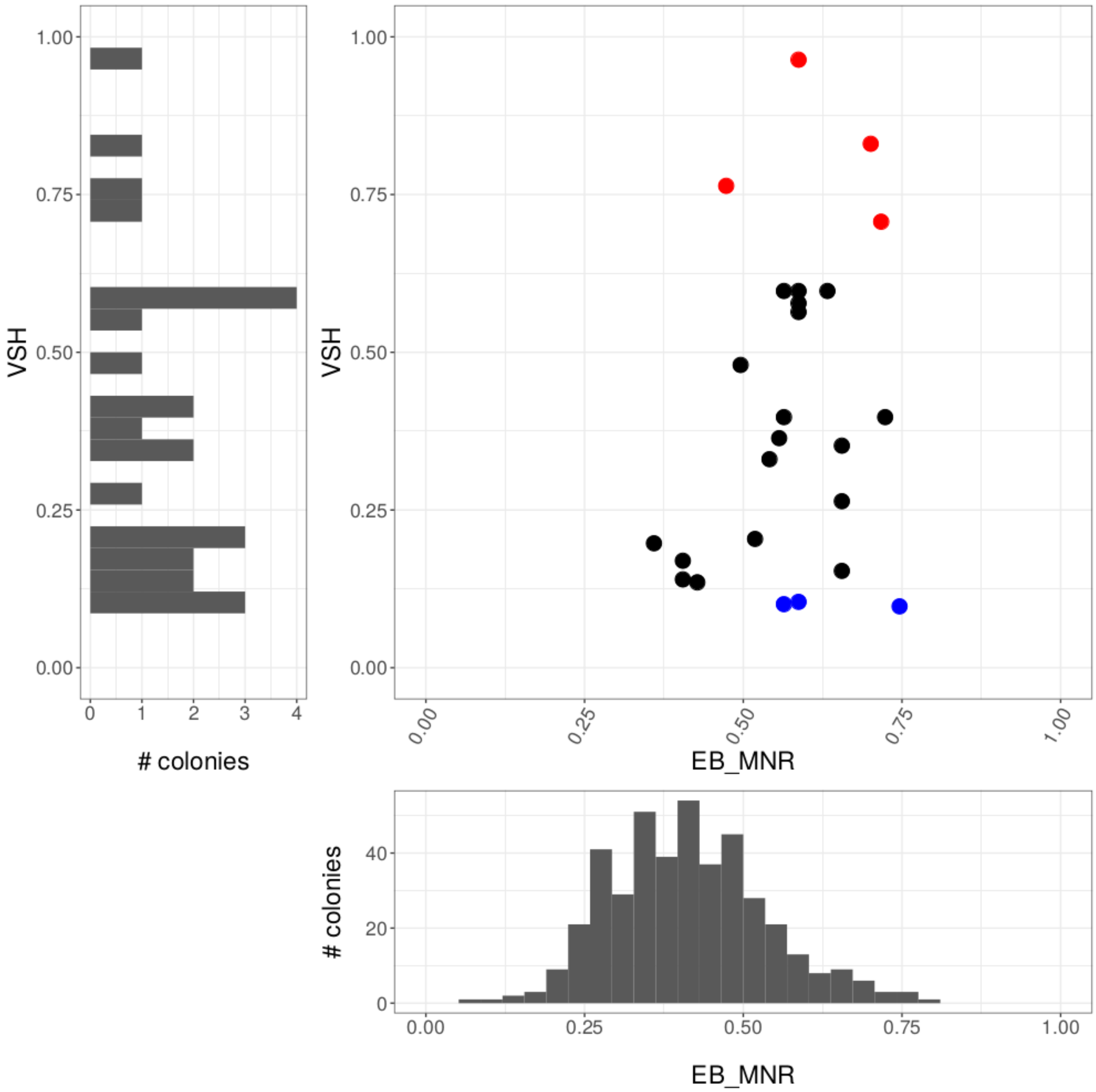

Table S1: Number of colonies in each of the different experiments (in bold) and number of colonies additionally contributing to other experiments. In square brackets, number of colonies used only in one experiment. In brackets and italic, number of apiaries on which colonies are placed. MNR: Mite Non Reproduction, HYG: Hygienic Behaviour and VSH: Varroa Sensitive Hygiene

	**Empirical Bayes MNR**	**Variance MNR**	**Short-term repeatibility MNR**	**Long-term repeatibility MNR**	**Repeatibility HYG**	**VSH**	**Model MNR**	**Model HYG**
**Empirical Bayes MNR**	**229** [0] *(14)*	35	31	55	125	25	229	136
**Variance MNR**	35	**39** [4] *(3)*	2	0	0	0	35	0
**Short-term repeatibility MNR**	31	2	**31** [0] *(NA)*	0	0	0	31	0
**Long-term repeatibility MNR**	55	0	0	**55** [0] *(9)*	50	9	55	53
**Repeatibility HYG**	125	0	0	50	**139** [0] *(9)*	12	127	139
**VSH**	25	0	0	9	12	**26** [1] *(1)*	25	12
**Model MNR**	229	35	31	55	127	25	**231** [0] *(14)*	138
**Model HYG**	136	0	0	55	139	12	138	**175** [25] *(9)*
